# SphK2/S1P Promotes Metastasis of Triple-Negative Breast Cancer Through the PAK1/LIMK1/Cofilin1 Signaling Pathway

**DOI:** 10.3389/fmolb.2021.598218

**Published:** 2021-04-22

**Authors:** Weiwei Shi, Ding Ma, Yin Cao, Lili Hu, Shuwen Liu, Dongliang Yan, Shan Zhang, Guang Zhang, Zhongxia Wang, Junhua Wu, Chunping Jiang

**Affiliations:** ^1^Department of Hepatobiliary Surgery, The Affiliated Drum Tower Hospital of Nanjing University Medical School, Nanjing, China; ^2^Jiangsu Key Laboratory of Molecular Medicine, Medical School, Nanjing University, Nanjing, China

**Keywords:** sphingosine kinase 2, sphingosine-1-phosphate, ABC294640, metastasis, triple-negative breast cancer

## Abstract

**Background:**

Triple-negative breast cancer (TNBC) features a poor prognosis, which is partially attributed to its high metastatic rate. However, there is no effective target for systemic TNBC therapy due to the absence of estrogen, progesterone, and human epidermal growth factor 2 receptors (ER, PR, and HER-2, respectively) in cancer. In the present study, we evaluated the role of sphingosine kinase 2 (SphK2) and its catalyst sphingosine-1-phosphate (S1P) in TNBC metastasis and the effect of the SphK2-specific inhibitor ABC294640 on TNBC metastasis.

**Methods:**

The function of SphK2 and S1P in TNBC cell metastasis was evaluated using transwell migration and wound-healing assays. The molecular mechanism of SphK2/S1P mediating TNBC metastasis was investigated using Western blot, histological examination, and immunohistochemistry assays. The antitumor activity of ABC294640 was examined in an *in vivo* TNBC lung metastatic model.

**Results:**

Sphingosine kinase 2 promoted TNBC cell migration through the generation of S1P. Targeting SphK2 with ABC294640 inhibited TNBC lung metastasis *in vivo*. p21-activated kinase 1 (PAK1), p-Lin-11/Isl-1/Mec-3 kinase 1 (LIMK1), and Cofilin1 were the downstream signaling molecules of SphK2/S1P. Inhibition of PAK1 suppressed SphK2/S1P-induced TNBC cell migration.

**Conclusion:**

Sphingosine kinase 2/sphingosine-1-phosphate promotes TNBC metastasis through the activation of the PAK1/LIMK1/Cofilin1 signaling pathway. ABC294640 inhibits TNBC metastasis *in vivo* and could be developed as a novel agent for the clinical treatment of TNBC.

## Introduction

Breast cancer is the most common malignant tumor and the leading cause of cancer-related death among women worldwide (Bray et al., 2018). Triple-negative breast cancer (TNBC) is a unique subtype of breast cancer in which the estrogen receptor (ER), progesterone receptor (PR), and human epidermal growth factor receptor 2 (HER-2) are not expressed (Perou et al., 2000). Although TNBC accounts for only 15–20% of breast cancers, it is characterized by profound invasion, poor prognosis, and short survival time. Moreover, patients with TNBC cannot receive endocrine and targeted therapies due to the lack of ER, PR, and HER-2 (Hwang et al., 2019). Finding new targets for TNBC treatment is of great clinical significance for patients with TNBC.

Accumulating evidence suggests that sphingosine-1-phosphate (S1P) is a potent bioactive lipid mediator that is involved in cancer development and progression by regulating tumor proliferation, migration, and angiogenesis (Hla and Brinkmann, 2011). Sphingosine kinase (SphK) is the key regulatory enzyme, catalyzing the formation of S1P. To date, two isoforms of SphK have been identified: SphK1 and SphK2 (Hait et al., 2006). The cancer-promoting functions of SphK1/S1P in TNBC are well-defined by compelling evidence. Previous studies have reported that SphK1/S1P promotes TNBC metastasis through the Notch signaling pathway (Wang et al., 2018) and that the inhibition of SphK1 reduces TNBC cell growth through the ERK1/2 and AKT signaling pathways (Datta et al., 2014). However, the role of SphK2/S1P in these processes is not clearly recognized.

Although early studies proposed a possible proapoptotic/anticancer function of SphK2 (Liu et al., 2003), accumulating evidence suggests that SphK2/S1P has tumor-promoting activity similar to SphK1/S1P. Qiu et al. (2016) reported that SphK2 promotes cell growth, migration, and invasion in papillary thyroid carcinoma. Knockdown of SphK2 inhibits the growth of human osteosarcoma cells (Xu et al., 2017). Moreover, ABC294640, a selective inhibitor of SphK2, was shown to suppress the progression of many cancers (French et al., 2010). Importantly, ABC294640 is currently under evaluation in a phase II clinical trial as an agent for the treatment of advanced hepatocellular carcinoma. The role of SphK2 in breast cancer has also been explored in recent studies. Antoon et al. (2011) reported that the SphK2 expression is higher in TNBC cells than in human breast epithelial cells, and high level of SphK2/S1P improved TNBC cell growth. In addition, the pharmacological inhibition of SphK2 slowed TNBC cell proliferation *in vitro* and *in vivo* (Antoon et al., 2010). However, few studies have focused on the effect of SphK2 on TNBC cell migration. Only Gao and Smith (2011) reported that the ablation of SphK2 inhibited the migration of MDA-MB-231 TNBC cells, but the underlying mechanism and whether ABC294640 could reduce the metastasis of TNBC have not been well elucidated.

Sphingosine-1-phosphate can promote breast cancer metastasis by activating multiple signaling cascades. S1P has been reported to increase p21-activated kinase 1 (PAK1) activity (Egom et al., 2010, 2011) and even directly stimulate PAK1 (Maceyka et al., 2008). In the presence of active PAK1, the phosphorylation of both p-Lin-11/Isl-1/Mec-3 kinase 1 (LIMK1) and Cofilin1 is greatly enhanced, which leads to actin cytoskeleton formation and increase in cell motility (Jang et al., 2012). Given the importance of PAK1 (Shrestha et al., 2012) and LIMK1 (Li et al., 2014) in breast cancer metastasis, we examined whether the PAK1/LIMK1/Cofilin1 signaling pathway is the downstream signaling cascade of SphK2/S1P.

In the present study, we explored the role and potential molecular mechanism of SphK2/S1P in TNBC metastasis. Moreover, ABC294640 was used to examine the effect of targeting SphK2 on TNBC metastasis.

## Results

### Knockdown of SphK2 Suppresses TNBC Cell Migration

**FIGURE 1:**
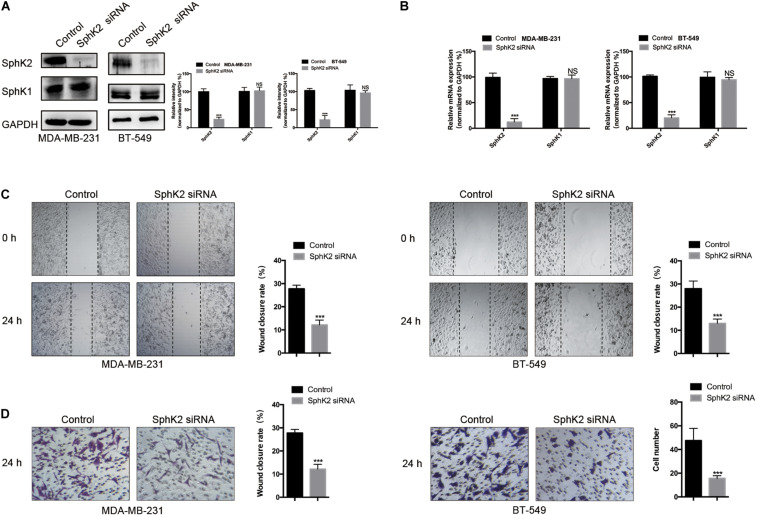
Effects of SphK2 knockdown on TNBC cell migration. **(A)** SphK2 and SphK1 protein expression in TNBC cells transfected with SphK2 siRNA or control siRNA was determined by Western blot assay. **(B)** Relative SphK2 and SphK1 mRNA expression determined by RT-qPCR in TNBC cells transfected with SphK2 siRNA or control siRNA. **(C)** The migration of TNBC cells transfected with SphK2 siRNA or control siRNA was evaluated by a wound-healing assay. **(D)** The migration of TNBC cells transfected with SphK2 siRNA or control siRNA was evaluated by a transwell assay. The results of each assay are representative of three independent experiments. The bars represent the mean ± SD of three independent experiments. ***p < 0.001; NS, not significant.

To investigate the role of SphK2 in TNBC cell migration, SphK2 siRNAs were transfected into two TNBC cell lines: MDA-MB-231 and BT-549. The successful knockdown of SphK2 in MDA-MB-231 and BT-549 cells was verified by real-time quantitative PCR (RT-qPCR) and Western blot assays. SphK2 siRNA transfection resulted in significantly decreased expression of SphK2 at both the mRNA and protein levels in MDA-MB-231 and BT-549 cells, whereas SphK1 expression was not affected (Figures 1A,B). Wound-healing and transwell migration assays were used to observe cell migration. The cell migration in the SphK2 siRNA groups was markedly lower than that in the control groups (Figures 1C,D), indicating that SphK2 plays an important role in TNBC cell migration.

### Pharmacological Inhibition of SphK2 Decreases TNBC Cell Migration

**FIGURE 2:**
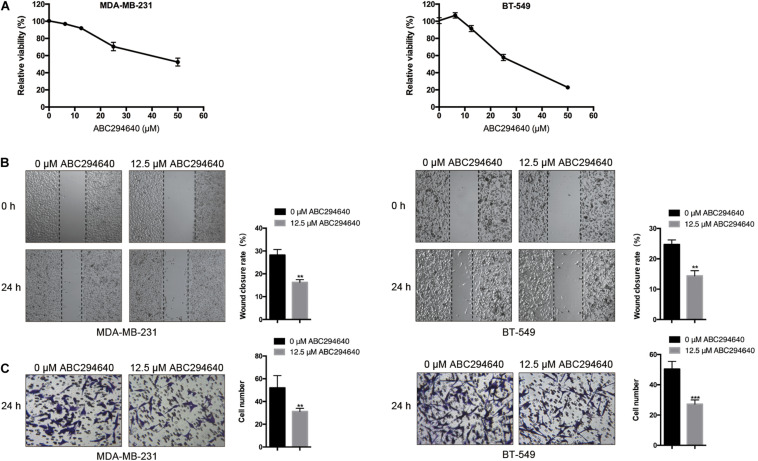
Effects of SphK2 inhibition on TNBC cell migration. **(A)** The effect of ABC294640 on TNBC cell viability was evaluated by a CCK-8 assay. **(B)** Wound-healing assay on the migration of TNBC cells treated with the SphK2 inhibitor ABC294640 (12.5 μM). **(C)** Transwell assay on the migration of TNBC cells exposed to ABC294640 (12.5 μM). The results of each assay are representative of three independent experiments. The bars represent the mean ± SD of three independent experiments. **p < 0.01, ***p < 0.001.

Sphingosine kinase 2 was inhibited with ABC294640, a selective SphK2 inhibitor, to assess the effect of SphK2 on TNBC cell migration. The CCK-8 assay was used to evaluate the effect of ABC294640 on TNBC cell viability, and a concentration of 12.5 μM was selected for the SphK2 inhibition experiment because no obvious inhibition of cell viability was observed at this concentration: relative cell viability was 91.62% for MDA-MB-231 cells and 90.76% for BT-549 cells (Figure 2A). The wound-healing and transwell migration assays showed that the migratory ability of both MDA-MB-231 and BT-549 cells was decreased after exposure to 12.5 μM ABC294640 for 24 h (Figures 2B,C).

### Sphingosine Kinase 2 Overexpression Promotes TNBC Cell Migration

**FIGURE 3:**
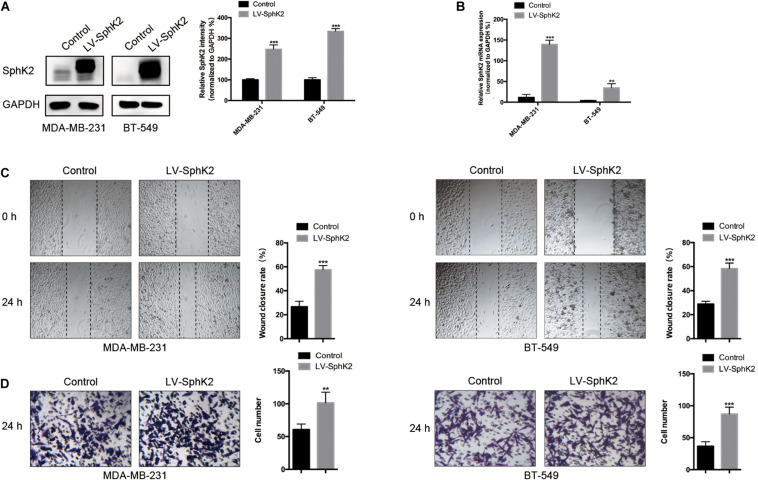
Effects of SphK2 overexpression on TNBC cell migration. **(A)** SphK2 protein expression in TNBC cells transfected with LV-SphK2 lentivirus or control virus was measured by Western blot assay. **(B)** Relative SphK2 mRNA expression levels in TNBC cells transfected with LV-SphK2 lentivirus or control virus were measured by RT-qPCR. **(C,D)** The migration of TNBC cells transfected with LV-SphK2 lentivirus or control lentivirus was evaluated by wound-healing and transwell assays. The results of each assay are representative of three independent experiments. The bars represent the mean ± SD of three independent experiments. **p < 0.01, ***p < 0.001.

MDA-MB-231 and BT-549 TNBC cells were stably transfected with LV-SphK2 lentivirus to enhance SphK2 expression. SphK2 expression in MDA-MB-231 and BT-549 cells was significantly increased compared with that in control cells confirmed by Western blot and qRT-PCR assays (Figures 3A,B). The migratory ability of LV-SphK2 TNBC cells was significantly increased, further supporting the importance of SphK2 in TNBC cell migration (Figures 3C,D).

### Sphingosine-1-Phosphate Production Positively Correlates With SphK2 Expression in TNBC Cells

**FIGURE 4:**
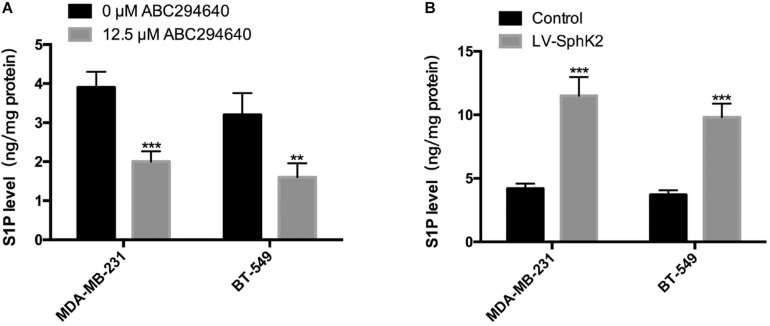
Influence of SphK2 pharmacological inhibition and overexpression on S1P production in TNBC cells. **(A)** Intracellular S1P production measured via LC-MS/MS in TNBC cells exposed to ABC294640 (12.5 μM) for 24 h. **(B)** S1P analyses were conducted in SphK2-overexpressing and control TNBC cells via LC-MS/MS. The results of each assay are representative of three independent experiments. The bars represent the mean ± SD of three independent experiments. **p < 0.01, ***p < 0.001.

Since the main biological function of SphK2 is to catalyze the generation of S1P, we further evaluated the role of S1P in SphK2-induced TNBC cell migration. The S1P level in TNBC cells after the inhibition or overexpression of SphK2 was measured by liquid chromatography-tandem mass spectrometry (LC-MS/MS). As expected, the pharmacological inhibition of SphK2 by ABC294640 decreased S1P production in MDA-MB-231 and BT-549 cells (Figure 4A; lowered by 0.49 and 0.5 compared with the control group, respectively). Meanwhile, overexpression of SphK2 increased the S1P level in these two cell lines (Figure 4B; 1.74-fold and 1.65-fold higher than control group, respectively). These data suggested that S1P production in TNBC cells was positively correlated with SphK2.

### Exogenous S1P Promotes TNBC Cell Migration and Restores the Reduced Migratory Ability of SphK2-Knockdown TNBC Cells

**FIGURE 5:**
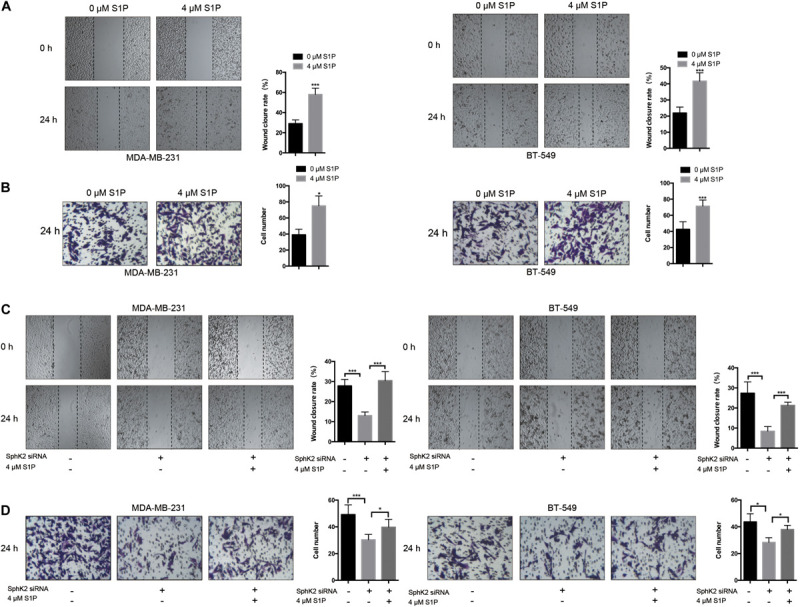
Effects of exogenous S1P on TNBC cell migration. **(A,B)** TNBC cell migration was examined after treatment with 4 μM S1P by wound-healing and transwell assays. **(C,D)** TNBC cells transfected with SphK2 siRNA were exposed to 4 μM S1P. Cell migration was evaluated by wound-healing and transwell assays. The results of each assay are representative of three independent experiments. The bars represent the mean ± SD of three independent experiments. *p < 0.05, ***p < 0.001.

Since the inhibition of SphK2 suppressed the S1P production, and SphK2 overexpression promoted S1P production in TNBC cells, we hypothesized that the effect of SphK2 on the migratory ability of TNBC cells was achieved by S1P. To determine whether S1P could promote the migration of TNBC cells, 4 μM exogenous S1P was added to the cell culture medium during transwell migration and wound-healing assays. Exogenous S1P stimulation markedly improved the migratory ability of TNBC cells (Figures 5A,B), suggesting that S1P promotes TNBC cell migration. Furthermore, when S1P was added to the medium of SphK2-knockdown TNBC cells, the impaired migratory ability was restored (Figures 5C,D). Collectively, the above results indicated that SphK2 promoted TNBC cell migration by catalyzing the production of S1P.

### Pharmacological Inhibition of PAK1 Decreases TNBC Cell Migration and Reduces the Increased Migratory Ability of TNBC Cells Due to SphK2 Overexpression or Exogenous S1P Stimulation

**FIGURE 6:**
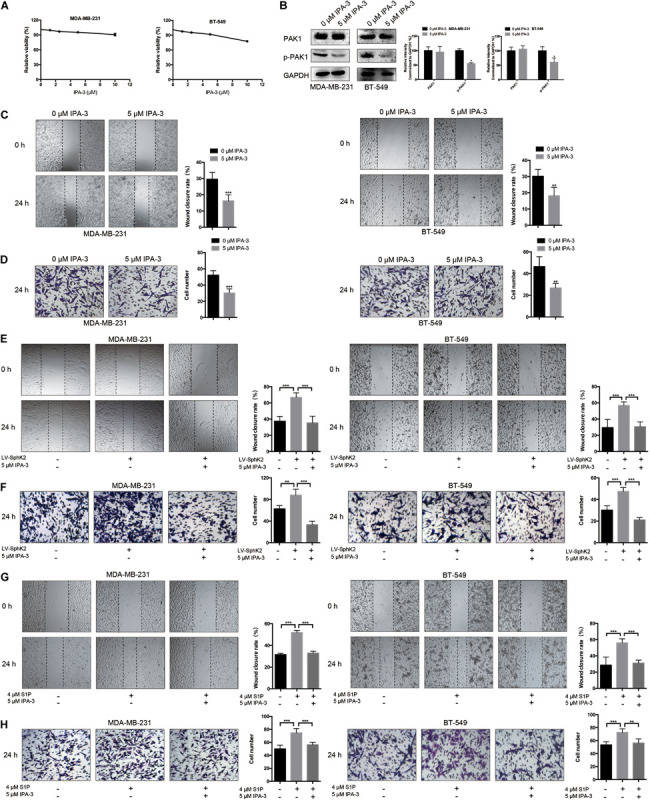
Effects of IPA-3 on TNBC cell migration. **(A)** The influence of IPA-3 on TNBC cell viability was evaluated by a CCK-8 assay. **(B)** The level of phosphorylated PAK1 protein in TNBC cells treated with 5 μM IPA-3 for 24 h was measured by Western blot assay. **(C,D)** TNBC cell migration was examined after treatment with 5 μM IPA-3 by wound-healing and transwell assays. **(E,F)** SphK2-overexpressing TNBC cells were exposed to 5 μM IPA-3, and migration was evaluated. **(G,H)** TNBC cells treated with S1P were exposed to 5 μM IPA-3, and migration was evaluated. The results of each assay are representative of three independent experiments. The bars represent the mean ± SD of three replications of experiments. *p < 0.05, **p < 0.01, ***p < 0.001.

Based on some research that S1P could promote cell migration through PAK1 activation, we questioned whether PAK1 was activated by SphK2/S1P in TNBC cells. To clarify the role of PAK1 in modulating TNBC metastasis, TNBC cells were exposed to the PAK1 inhibitor IPA-3, and a concentration of 5 μM was selected based on the PAK1 inhibition experiment, in which viability of cells exposed to 5 μM IPA-3 was 94.70% in MDA-MB-231 cells and 90.77% in BT-549 cells, showing no significant inhibition of cell viability (Figure 6A). After exposure to 5 μM IPA-3 for 24 h, PAK1 phosphorylation declined (Figure 6B), indicating that PAK1 activity was decreased. The results of the transwell migration and wound-healing assays showed that the inhibition of PAK1 activity by IPA-3 decreased the migration of TNBC cells (Figures 6C,D). In addition, the increased migration ability of TNBC cells due to SphK2 overexpression or exogenous S1P stimulation was reversed by IPA-3 treatment (Figures 6E–H). Therefore, PAK1 was confirmed to promote the migration of TNBC cells and could be a downstream molecule of SphK2/S1P.

### Sphingosine Kinase 2/Sphingosine-1-Phosphate Regulates TNBC Cell Migration Through the Activation of PAK1/LIMK1/Cofilin1 Signaling

**FIGURE 7:**
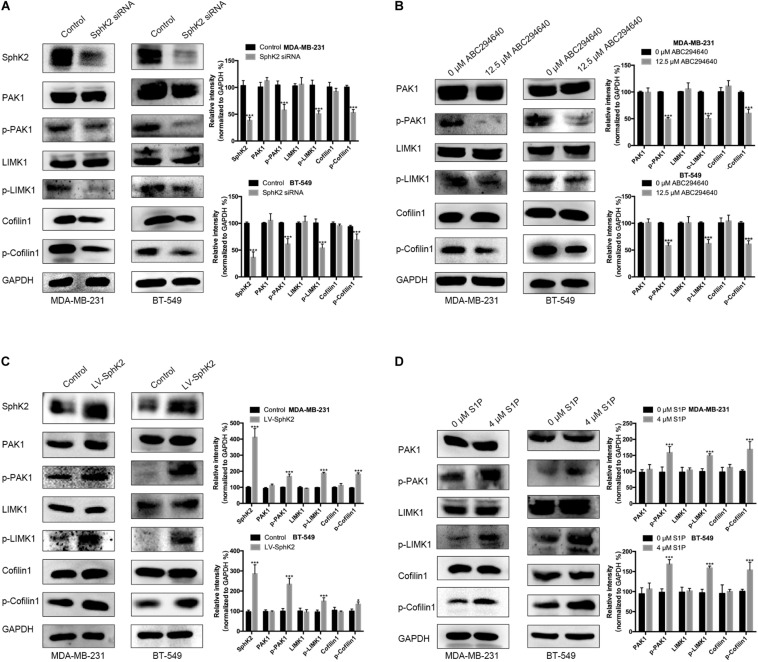
The phosphorylation of PAK1, LIMK1, and Cofilin1 in different cell groups. Western blot assay on **(A)** the phosphorylation of PAK1, LIMK1, and Cofilin1 in TNBC cells transfected with SphK2 siRNA. **(B)** The phosphorylation of PAK1, LIMK1, and Cofilin1 in TNBC cells treated with ABC294640. **(C)** The phosphorylation of PAK1, LIMK1, and Cofilin1 in SphK2-overexpressing TNBC cells. **(D)** The phosphorylation of PAK1, LIMK1, and Cofilin1 in TNBC cells exposed to S1P. The results of each assay are representative of three independent experiments. The bars represent the mean ± SD of three replications of experiments. *p < 0.05, ***p < 0.001.

Currently, the molecular mechanisms of SphK2-mediated TNBC cell migration are unknown. Since PAK1 is a downstream molecule of SphK2/S1P, and PAK1 can promote cell motility through the activation of LIMK1/Cofilin1, we hypothesized that SphK2/S1P could regulate the migration of TNBC cells in a PAK1/LIMK1/Cofilin1 signaling-dependent manner. Therefore, we measured the phosphorylation level of PAK1, LIMK1, and Cofilin1 in TNBC cells subjected to different treatments. The Western blot assay results showed that the phosphorylation of PAK1, LIMK1, and Cofilin1 was decreased in TNBC cells with SphK2 knockdown or inhibition (Figures 7A,B) but increased in SphK2-overexpressed TNBC cells (Figure 7C). The exogenous S1P stimulation also increased the phosphorylation of PAK1, LIMK1, and Cofilin1 (Figure 7D). These results suggested that the PAK1/LIMK1/Cofilin1 signaling pathway participates in SphK2/S1P-mediated migration of MDA-MB-231 and BT-549 cells.

### Sphingosine Kinase 2-Selective Inhibitor ABC294640 Reduces the Lung Metastasis of TNBC Cells *in vivo*

**FIGURE 8:**
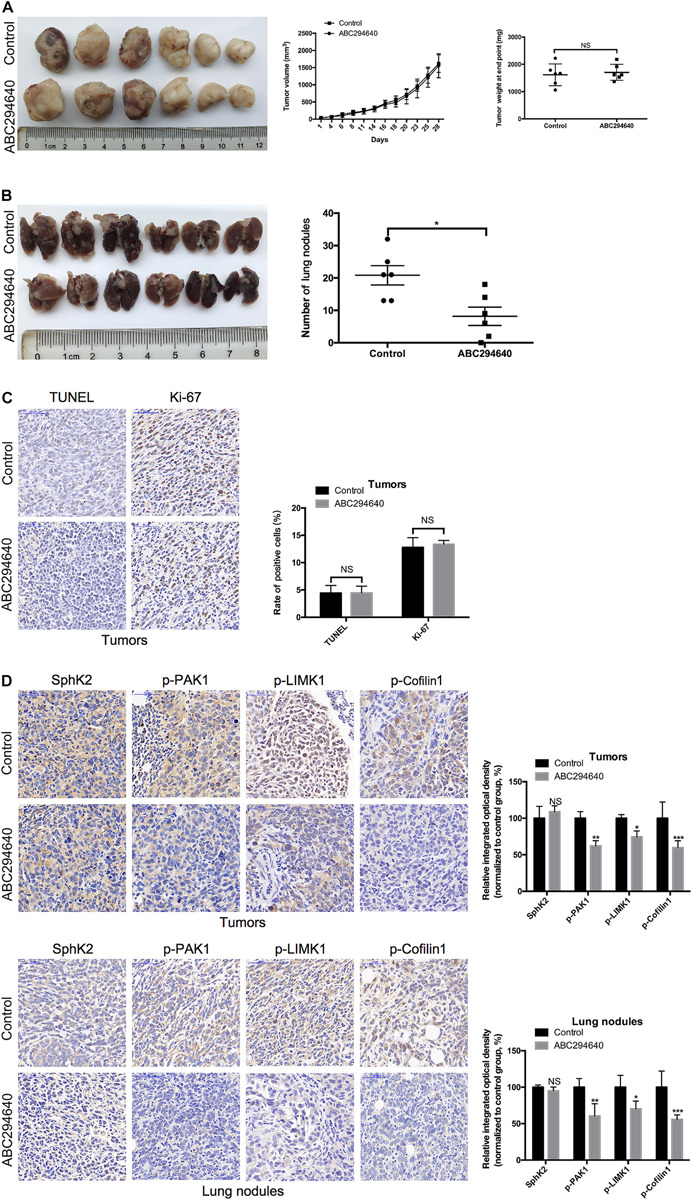
Inhibition of SphK2 activity reduces TNBC metastasis and the phosphorylation of PAK1, LIMK1, and Cofilin1 *in vivo*. **(A)** Representative images of the orthotopic tumors were obtained, and the volumes and weight of tumors were recorded. **(B)** Representative images of the lungs were obtained, and the metastatic nodules were counted. **(C)** Tumor apoptosis and proliferation were evaluated by TUNEL and Ki-67 staining, respectively. **(D)** The levels of SphK2 and phosphorylated PAK1, LIMK1, and Cofilin1 in orthotopic tumors and lung metastatic nodules were measured. The bars represent the mean ± SD of replications of experiments. *p < 0.05, **p < 0.01, ***p < 0.001; NS, not significant.

To determine the therapeutic potential of pharmacological inhibition of SphK2 in TNBC, the effect of ABC294640 on tumor metastasis was examined in a 4T1 xenograft mouse model. Mice with established, size-matched 4T1 tumors were divided into two groups and treated with ABC294640 or vehicle. Tumor weight showed no significant difference between groups at the end point of treatment (Figure 8A). There was also no distinct difference in the tumor volume between the two groups during 4 weeks of treatment (Figure 8A), while an obvious increase in the number and size of lung metastatic nodules was observed in the vehicle control group at the end of the treatment (Figure 8B), indicating that ABC294640 could decrease TNBC metastasis *in vivo*. Moreover, we performed a histological assessment on orthotopic tumors and lung metastatic nodules. No significant difference in TUNEL or Ki-67 staining was observed between tumors from ABC294640-treated mice and those from control mice, indicating that treatment with 40 mg/kg ABC294640 had minimal influence on tumor apoptosis or proliferation (Figure 8C). Similar to the results *in vitro*, orthotopic tumors and lung metastatic nodules from the control group exhibited stronger staining for p-PAK1, p-LIMK1, and p-Cofilin1 than that from ABC2945640-treated group (Figure 8D), further supporting the hypothesis that SphK2/S1P regulates the metastasis of TNBC through the activation of PAK1/LIMK1/Cofilin1 signaling pathway.

## Discussion

Triple-negative breast cancer is a highly aggressive cancer that lacks targeted therapy (De Laurentiis et al., 2010); therefore, the need to identify efficient targets for TNBC therapy is important. Our research showed that SphK2, a key enzyme that converts sphingosine to S1P, is involved in TNBC cell migration. Similar to the results observed in human renal cancer cells (Gao and Smith, 2011), the ablation of SphK2 decreased TNBC cell migration. In addition, the pharmacological inhibition of SphK2 with ABC294640 diminished TNBC cell migration in the present study. Another study reported that the inhibition of SphK2 activity by other inhibitors had an inhibitory effect on the migration in HeLa cells (Lee et al., 2017). Moreover, we found that the overexpression of SphK2 led to an increased migratory ability of TNBC cells. Our results were in consistent with a previous study that reported that upregulation of SphK2 partially increased the migration of papillary thyroid carcinoma cells (Qiu et al., 2016). The results from the present study and others suggest that SphK2 is involved in tumor metastasis and that SphK2 might be a therapeutic target in TNBC.

Accumulating evidence demonstrates that S1P is a critical second messenger that regulates the migration of various cells, including cancer cells (Sekine et al., 2011; Bao et al., 2012) myofibroblasts (Li et al., 2012), dendritic cells (Eigenbrod et al., 2006), and stem cells (Ng et al., 2018). Moreover, it has been reported that S1P generated from SphK1 accelerates breast cancer cell migration (Nagahashi et al., 2018). However, whether SphK2/S1P plays the same role in TNBC cell migration is unclear. Here, we reported that SphK2/S1P promotes the migration of TNBC cells. We demonstrated that the inhibition of SphK2 with ABC294640 reduced the S1P level and that the overexpression of SphK2 increased the S1P level in TNBC cells, indicating that the expression of S1P is positively correlated with SphK2. The positive correlation between SphK2 and S1P also existed in murine adenocarcinoma cells (French et al., 2010) and colorectal cancer cells (Xun et al., 2015). However, Gao and Smith (2011) reported contrasting results in which knockdown of SphK2 led to increased S1P production in renal carcinoma cells owing to the elevated expression of SphK1. In the present study, SphK2 knockdown had minimal influence on SphK1 expression. Hait et al. (2005) also showed that SphK2 knockdown did not affect the expression and activity of SphK1 in breast cancer cells. We showed that exogenous S1P stimulation promoted TNBC cell migration and SphK2 knockdown reduced the migratory ability of TNBC cells. Collectively, these results suggest that SphK2 promotes TNBC cell migration through the production of S1P.

Based on the important roles of SphK2/S1P in TNBC cell migration, we further investigated the downstream molecular mechanisms. Our data showed that the PAK1/LIMK1/Cofilin1 cascade, an important regulator of actin cytoskeleton and cell motility (Dummler et al., 2009), was activated by SphK2/S1P in TNBC cells. S1P has been reported to increase PAK1 activity (Egom et al., 2011) and even directly stimulate PAK1 to induce cell lamellipodia formation and movement (Maceyka et al., 2008). Consistent with these studies, we found that exogenous S1P stimulation increased the phosphorylation of PAK1. In addition, the inhibition of PAK1 by IPA-3 decreased TNBC cell migration and reversed the increased migratory ability of TNBC cells due to SphK2 overexpression or S1P stimulation. These results indicate that PAK1 is the downstream target of SphK2/S1P and contributes to the migration of TNBC cells. Furthermore, the phosphorylation of PAK1, LIMK1, and Cofilin1 was increased in SphK2-overexpressed or S1P-stimulated TNBC cells and decreased in SphK2-inhibited TNBC cells, and was even decreased *in vivo* due to the administration of ABC294640. These results demonstrated that the PAK1/LIMK1/Cofilin1 cascade is the downstream signaling pathway of SphK2/S1P. Generally, we elucidate a novel mechanism linking SphK2/S1P to PAK1/LIMK1/Cofilin1 in TNBC cell migration.

ABC294640, the selective inhibitor of SphK2, was found to have broad antitumor activity. ABC294640 inhibited cell growth both *in vitro* and *in vivo* in various cancers such as colorectal cancer (Xun et al., 2015), non-small cell lung cancer (Dai et al., 2018), and prostate cancer (Schrecengost et al., 2015). ABC294640 also has an effective inhibitory effect on cancer cell migration *in vitro* (French et al., 2010). Remarkably, the present study provided the first evidence that the pharmacological inhibition of SphK2 by ABC294640 at a dose of 40 mg/kg decreased TNBC metastasis in a mouse model, indicating the clinical value of ABC294640 for the treatment of TNBC.

Several limitations of this present study should be noted. Although Antoon et al. (2011) reported that the expression of SphK2 in TNBC cell line MDA-MB-231 was significantly higher than in normal mammary epithelial cell MCF-10A, the evidence of whether SphK2 upregulation present in TNBC tissues is still lacking. Our current study also did not provide expression data of SphK2 in human TNBC tissues, which should be considered as a limitation of the study. Further investigations based on clinical specimens are warranted to provide more evidence supporting SphK2 as a therapeutic target of TNBC. Pharmacokinetics and pharmacodynamics data of ABC294640 in tumor-bearing mice and in patients with advanced solid tumors are available in previously published reports (French et al., 2010; Britten et al., 2017). Interestingly, our results showed that the oral administration of 40 mg/kg ABC294640 three times a week exhibited dramatic activity against TNBC metastasis without inhibiting primary tumor growth. Unfortunately, the plasmatic and intratumoral concentrations of ABC294640 in this metastatic model remain unknown, which is also a limitation of this study. The determination of drug concentrations at which ABC294640 demonstrates metastasis-specific activity will be of great significance in further studies to develop therapeutic strategies against TNBC metastasis.

## Conclusion

Collectively, we reported that SphK2/S1P promotes TNBC cell migration through the activation of the PAK1/LIMK1/Cofilin1 signaling pathway. Targeting SphK2 with ABC294640 inhibits TNBC xenograft metastasis *in vivo*, and ABC294640 has the potential to be a novel agent for the clinical treatment of TNBC.

## Materials and Methods

### Cell Culture

Human breast carcinoma cell lines MDA-MB-231 and BT-549 and the mouse breast cancer cell line 4T1 were purchased from the Cell Bank of the Chinese Academy of Sciences (Shanghai, China). MDA-MB-231 cells were cultured in Dulbecco’s modified Eagle’s medium (DMEM) supplemented with 10% (v/v) fetal bovine serum (FBS), 100 U/ml penicillin, and 100 μg/ml streptomycin (all from Wisent, St-Bruno, Canada). BT-549 and 4T1 cells were cultured in Roswell Park Memorial Institute-1640 (RPMI-1640; Wisent, St-Bruno, Canada) medium, and other components of the culture media were the same as for MDA-MB-231 cells. Cells were cultured at 37°C in a humidified 5% CO_2_ atmosphere.

### Small Interfering RNA Transfection

Sphingosine kinase 2 was downregulated by transfection with sequence-specific siRNA (GenePharma, Shanghai, China). siRNA against human SphK2 (targeted sequence: 5′ GGGUAGUGCCUGAUCAAUGTT 3′) and control siRNA were used. A total of 4 μl of Lipofectamine 2000 (Thermo Fisher Scientific, Waltham, MA, United States) was mixed with 150 μl of Opti-MEM (Wisent, St-Bruno, Canada) and incubated for 5 min at room temperature. siRNA was diluted in 150 μl of Opti-MEM. Following 5 min of incubation, the diluted siRNA was combined with diluted Lipofectamine 2000 (total volume, 300 μl). The solution was mixed gently, incubated for 20 min at room temperature, and then added to a six-well dish containing cells and medium. RT-qPCR and Western blot assays were adopted to assess the knockdown efficiency.

### Lentivirus Transfection

Lentivirus transfection was used to obtain TNBC cells with stable ectopic SphK2 expression. Lentivirus expressing SphK2 and corresponding negative control virus were purchased from GeneChem (Shanghai, China). TNBC cells were plated in the six-well plates at a density of 2 × 10^5^ cells per well and were subsequently transfected with lentivirus at a multiplicity of infection (MOI) of 10. Following 48 h of incubation, the antibiotic-resistant transfected cells were selected by applying a culture medium containing puromycin. The SphK2 overexpression efficiency was confirmed by Western blot and RT-qPCR assays.

### Cell Counting Kit-8 Assay

For the Cell Counting Kit-8 (CCK-8) assay, the IPA-3 (Selleck, Houston, TX, United States), ABC294640 (Selleck, Houston, TX, United States), and S1P (Avanti Polar Lipids, Alabaster, AL, United States) were dissolved in dimethyl sulfoxide (DMSO) to generate stock solutions at concentrations of 50, 50, and 10 mM, respectively. The final concentration of DMSO in the treatment medium was below 0.1%. TNBC cells in DMEM containing 10% FBS were seeded into 96-well plates at a concentration of 1 × 10^4^ cells per well and incubated for 24 h. The culture medium was replaced with a fresh medium containing vehicle or testing agents at indicated concentrations. After treating cells with different agents or vehicles for 48 h, CCK-8 solution (10 μl/well) was added to the 96-well plates and incubated for 1 h to detect the viability of TNBC cells. The light absorbance values at 450 nm were measured in a microplate reader (Bio-Rad, Hercules, CA, United States), and cell viability was determined. Relative viability was normalized to the vehicle-treated control cells after background subtraction and was expressed as OD_*test*_/OD_*control*_^∗^100%. Each treatment was performed in triplicate wells, and three independent experiments were repeated.

### Protein Isolation and Western Blot Assays

The cells were lysed with 150 μl of lysis buffer (Beyotime, Shanghai, China) containing 1% protease inhibitors (Thermo Fisher Scientific, Waltham, MA, United States) on ice for 5 min following washing two times with ice-cold phosphate-buffered saline (PBS). The cells were harvested and centrifuged at 12,000 × *g* for 5 min at 4°C. The protein concentrations were determined using a BCA kit (Beyotime, Shanghai, China). Equal amounts of protein (20 μg/lane) dissolved in 20 μl of loading buffer (Beyotime, Shanghai, China) were separated by sodium dodecyl sulfate polyacrylamide gel electrophoresis (SDS-PAGE, Beyotime, Shanghai, China), transferred to polyvinylidene difluoride (PVDF) membranes (Roche Applied Science, Mannheim, Germany), and blocked with 5% non-fat milk for 1 h at room temperature. Immunoblotting was performed by incubation overnight at 4°C with the indicated primary antibodies (Cell Signaling Technology, Beverly, MA, United States except as noted): anti-PAK1, anti-p-PAK1, anti-Cofilin1, anti-p-Cofilin1, anti-LIMK1 (Abcam, Burlingame, CA, United States), anti-p-LIMK1 (Abcam, Burlingame, CA, United States), anti-SphK1 (Proteintech, Wuhan, China), and anti-SphK2 (Proteintech, Wuhan, China). The dilution of primary antibodies against SphK1 and SphK2 was 1:500. Other primary antibodies were diluted at 1:1,000. After the incubation with primary antibodies, the membranes were washed and incubated with horseradish peroxidase (HRP)-linked secondary antibodies (1:5,000 dilution; Proteintech, Wuhan, China) at room temperature for 1 h. The signals were developed with an enhanced chemiluminescence reagent (Biosharp, Beijing, China) under a chemiluminescence camera (Tanon, Beijing, China). The density of each band was measured using ImageJ software (National Institutes of Health, Bethesda, MD, United States) and normalized to internal control [glyceraldehyde 3-phosphate dehydrogenase (GAPDH)] from the same sample. Three independent experiments were repeated.

### Real-Time Quantitative PCR

Total RNA was extracted using the TRIzol Reagent (Takara Bio, Otsu, Japan) and reverse transcribed into cDNA using the PrimeScript RT Master Mix (Takara Bio, Otsu, Japan). The relative mRNA expression levels were determined by RT-qPCR with the SYBR Green PCR Master Mix (Takara Bio, Otsu, Japan) on an ABI PRISM 7300 Sequence Detection System (Applied Biosystems, Foster City, CA, United States). The relative mRNA levels were calculated by the 2^–ΔΔ^ *^*Cq*^* method with GAPDH as the internal control. Three independent experiments were repeated.

### Wound-Healing Assay

A culture insert (Ibidi, Munich, Germany) was used to generate a wound of 500 μm. The insert was placed on the 24-well plates; then, 2 × 10^5^ cells were seeded in each culture insert and incubated for 24 h. After removing the culture insert, cells were allowed to grow in the media without FBS for 24–48 h. The original area and migration area were measured using ImageJ software, and the wound closure rates are shown according to the ratio of the migration area to the original area. Each treatment was performed in triplicate wells and three independent experiments were repeated.

### Transwell Migration Assay

Transwell migration assay was performed using a 6.5-mm transwell insert with 8.0-μm pore polycarbonate membrane (Merck Millipore, Burlington, MA, United States). A total of 300 μl of cell suspension containing 2 × 10^5^ cells without FBS was added to the upper chamber, and 800 μl of medium containing 10% FBS was added to the lower chamber. After incubation for 48 h, cells on the lower chamber were fixed with 4% paraformaldehyde for 20 min and stained with crystal violet for 20 min. Images of each chamber were captured randomly for cell counting. Three independent experiments were repeated.

### Quantification of S1P by LC-MS/MS

Cells were washed two times with cold PBS, harvested, and centrifuged at 1,000 rpm for 5 min at 4°C; then, the cells were suspended in 100 μl of distilled water. The cell suspension was mixed with internal standard (1 ng/ml C17-S1P, Avanti Polar Lipids, Alabaster, AL, United States) and 65 μl of methanol and then centrifuged at 1,000 rpm for 5 min. S1P in the supernatant was quantified by LC-MS/MS as described previously at the Virginia Commonwealth University Lipidomics Core (Nagahashi et al., 2013).

### Tumor Xenograft Model

Six-week-old female BALB/c mice, weighing approximately 20 g, were purchased from the Model Animal Research Center of Nanjing University (Nanjing, China). The mice were housed in sterile cages in laminar airflow hoods in a specific pathogen-free environment at 22–25°C, 40–60% relative humidity with a 12:12 h day/night light cycle. The mice had free access to autoclaved water and commercial mouse chow (Xietong Biological, Nanjing, China). The study protocol was approved by the Institutional Ethics Committee of the Affiliated Drum Tower Hospital of Nanjing University Medical School. 4T1 cells (2 × 10^5^ cells in 100 μl of PBS) were surgically implanted into the mammary fat pads. When the tumors formed, the mice were randomly assigned into two groups. Subsequently, ABC294640 at an oral dose of 40 mg/kg body weight or vehicle was administered three times a week. ABC294640 was suspended in an oral vehicle solution containing 2% DMSO + 30% PEG300 (Selleck, Houston, TX, United States) + 5% Tween 80 (Selleck, Houston, TX, United States) + ddH_2_O. Tumor volume was measured three times a week using a digital caliper and calculated using the equation (length × width^2^)/2. The body weight of mice was also measured three times a week. All mice were sacrificed by cervical dislocation under general anesthesia with isoflurane (RWD Life Science, Shenzhen, China) after 4 weeks of treatment. The tumors and lungs were harvested, and the number of metastatic tumor nodules was recorded.

### Hematoxylin & Eosin (H&E), Immunohistochemical, and TUNEL Staining

Tissues fixed with 4% paraformaldehyde were embedded in paraffin and cut into 5-μm thick slices. For H&E staining, the tissue slices were dewaxed in xylene, rehydrated with decreasing concentrations of ethanol, and washed with PBS. The slices were stained with hematoxylin for 30 s with agitation and rinsed with water. Then, the slices were stained with eosin for 10–30 s with agitation and rinsed with water. After staining, the slices were dehydrated, mounted, and covered with coverslips. Immunohistochemical (IHC) staining was performed according to a published protocol (Liu et al., 2017). Samples were incubated with antibodies (Cell Signaling Technology, Beverly, MA, United States, except as noted) against Ki-67, SphK2 (Proteintech, Wuhan, China), p-PAK1, p-LIMK1 (Abcam, Burlingame, CA, United States), and p-Cofilin1. TUNEL staining was performed according to the manufacturer’s protocol (Beyotime Biotechnology, Nantong, China). The Ki-67-positive cells, TUNEL-positive cells, and the integrated optical density (IOD) of SphK2, p-PAK1, p-LIMK1, and p-Cofilin1 staining were analyzed using ImageJ software.

### Statistical Analysis

Data were analyzed using SPSS 19.0 statistical software (IBM, Chicago, IL, United States) and are expressed as the mean ± SD. Comparisons of different groups were performed using Student’s *t*-test or ANOVA analysis. A *p*-value less than 0.05 (*p* < 0.05) was considered to indicate a significant difference.

## Data Availability Statement

The raw data supporting the conclusions of this article will be made available by the authors, without undue reservation.

## Ethics Statement

The animal study was reviewed and approved by the Institutional Ethics Committee of the Affiliated Drum Tower Hospital of Nanjing University Medical School.

## Author Contributions

CJ, JW, ZW, and WS conceived and designed the experiments. WS, DM, YC, LH, SL, DY, SZ, and GZ performed the experiments. WS, DM, YC, LH, and SL analyzed the data. WS and SZ wrote the original manuscript. JW and ZW reviewed and edited the manuscript. CJ, JW, and ZW acquired the funding. All authors contributed to the article and approved the submitted version.

## Conflict of Interest

The authors declare that the research was conducted in the absence of any commercial or financial relationships that could be construed as a potential conflict of interest.
